# Lysosomal biogenesis and function in osteoclasts: a comprehensive review

**DOI:** 10.3389/fcell.2024.1431566

**Published:** 2024-08-07

**Authors:** Junchen Jiang, Rufeng Ren, Weiyuan Fang, Jiansen Miao, Zijun Wen, Xiangyang Wang, Jiake Xu, Haiming Jin

**Affiliations:** ^1^ Department of Orthopaedics, The Second Affiliated Hospital and Yuying Children’s Hospital of Wenzhou Medical University, Wenzhou, China; ^2^ The Second School of Medicine, Wenzhou Medical University, Wenzhou, China; ^3^ Shenzhen Institute of Advanced Technology, Chinese Academy of Sciences, Shenzhen, China

**Keywords:** lysosome, osteoclast, bone resorption, autophagy, bone metabolic disorders

## Abstract

Lysosomes serve as catabolic centers and signaling hubs in cells, regulating a multitude of cellular processes such as intracellular environment homeostasis, macromolecule degradation, intracellular vesicle trafficking and autophagy. Alterations in lysosomal level and function are crucial for cellular adaptation to external stimuli, with lysosome dysfunction being implicated in the pathogenesis of numerous diseases. Osteoclasts (OCs), as multinucleated cells responsible for bone resorption and maintaining bone homeostasis, have a complex relationship with lysosomes that is not fully understood. Dysregulated function of OCs can disrupt bone homeostasis leading to the development of various bone disorders. The regulation of OC differentiation and bone resorption for the treatment of bone disease have received considerable attention in recent years, yet the role and regulation of lysosomes in OCs, as well as the potential therapeutic implications of intervening in lysosomal biologic behavior for the treatment of bone diseases, remain relatively understudied. This review aims to elucidate the mechanisms involved in lysosomal biogenesis and to discuss the functions of lysosomes in OCs, specifically in relation to differentiation, bone resorption, and autophagy. Finally, we explore the potential therapeutic implication of targeting lysosomes in the treatment of bone metabolic disorders.

## 1 Highlights

• Lysosome biogenesis involves protein transport, lysosomal fusion and reformation, regulated by various factors.
• Lysosomes play a pivotal role in osteoclasts, modulating cellular processes and bone metabolism.
• Lysosomes contribute to osteoclast autophagy, affecting the bone resorption.
• Targeting lysosomes offers a potential therapeutic approach for bone metabolic disorders.

## 2 Introduction

Numerous bone disorders, including osteoporosis (Zhivodernikov et al., 2023), osteomalacia (Grünherz et al., 2020), rheumatoid arthritis (Hascoët et al., 2023), and periodontitis (Lin et al., 2022), are closely associated with abnormal OCs, which exhibit high bone resorption activity. These diseases have shared characteristics, including hyperactive and differentiated OCs with enhanced bone resorption activity. Additionally, the differentiation and bone resorption pathways serve as therapeutic targets for various pharmacological agents, such as bisphosphonates and denosumab, commonly employed in clinical practice. To be specific, bisphosphonates impair OC differentiation and resorptive activity by blocking the mevalonate pathway through the inhibition of farnesyl pyrophosphate synthase (Chin et al., 2022; Scala et al., 2022), whereas denosumab impedes OC differentiation by targeting RANKL (Reid and Billington, 2022; Ferrari and Langdahl, 2023). Nonetheless, these interventions lead to reduced bone formation as a consequence of the decoupling between OCs and OBs (Iñiguez-Ariza and Clarke, 2015). Therefore, a comprehensive comprehension of the molecular mechanisms governing OC differentiation and bone resorption function is imperative for the identification of novel therapeutic targets for such skeletal disorders.

Research on OC differentiation and bone resorption has been extensively explored, yet the investigation into lysosome biogenesis and function in OCs remains limited. Lysosomes, which are characterized by a single membrane and dynamic, heterogeneous nature, play crucial roles in degradation and metabolic regulation (Paudel et al., 2023; Cao et al., 2021; Ballabio and Bonifacino, 2020). As revealed via mechanistic studies, lysosomes serve as a regulatory hub for multiple vesicle trafficking pathways, including endocytic, phagocytic, and autophagic pathways, with specific functional proteins in the processes of bone resorption and autophagy (Nakanishi-Matsui and Matsumoto, 2022; Mahapatra et al., 2021). The acidic microenvironment formation and bone matrix degradation during bone resorption rely on the biogenesis and transport of lysosomes (Lacombe et al., 2013), while autophagy, a lysosome-mediated catabolic process, facilitates the degradation and recycling of cellular components, playing a pivotal role in bone metabolism by regulating osteoclast activity and lifespan. (Mahapatra et al., 2021). However, the specific mechanisms involved in the regulation and biogenesis of lysosomes in OCs remain to be further investigated. Therefore, we focus our review on the biogenesis of lysosomes and the regulation of lysosomes in OCs, including differentiation, bone resorption and autophagy phases.

## 3 Lysosomal constituents and biogenesis

### 3.1 Functional lysosomal protein

Lysosomes consist of an acidic lumen enclosed by a phospholipid bilayer plasma membrane. The acidic environment within the lumen is essential for physiological functions of lysosomes, which affect the activity of both membrane and luminal hydrolases (Xu and Ren, 2015; Zhang et al., 2023). The lysosomal hydrolases and membrane proteins, which serve as functional proteins within the lysosome, play a crucial role in maintaining lysosomal function.

#### 3.1.1 Lysosomal hydrolases

The acid lumen of lysosomes contains a variety of degradative enzymes (also called acid hydrolases), including glycosidases, proteases, nucleases, lipases, phosphatases, and phospholipases (Jain et al., 2022). Over 60 hydrolases have been identified, each with specific substrates for degradation, collectively determining the lysosome’s degradation capability. In addition to degrading cellular waste and processing pro-protein, lysosomal hydrolases also participate in the process of antigen processing, membrane repair, and the initiation of apoptosis (Mutvei et al., 2023; Yang et al., 2023; Pellegrini et al., 2023). In the bone resorption process of OCs, lysosomal hydrolases play a significant role. Information about the detailed hydrolases involved in bone resorption is discussed further in the last section.

**FIGURE 1:**
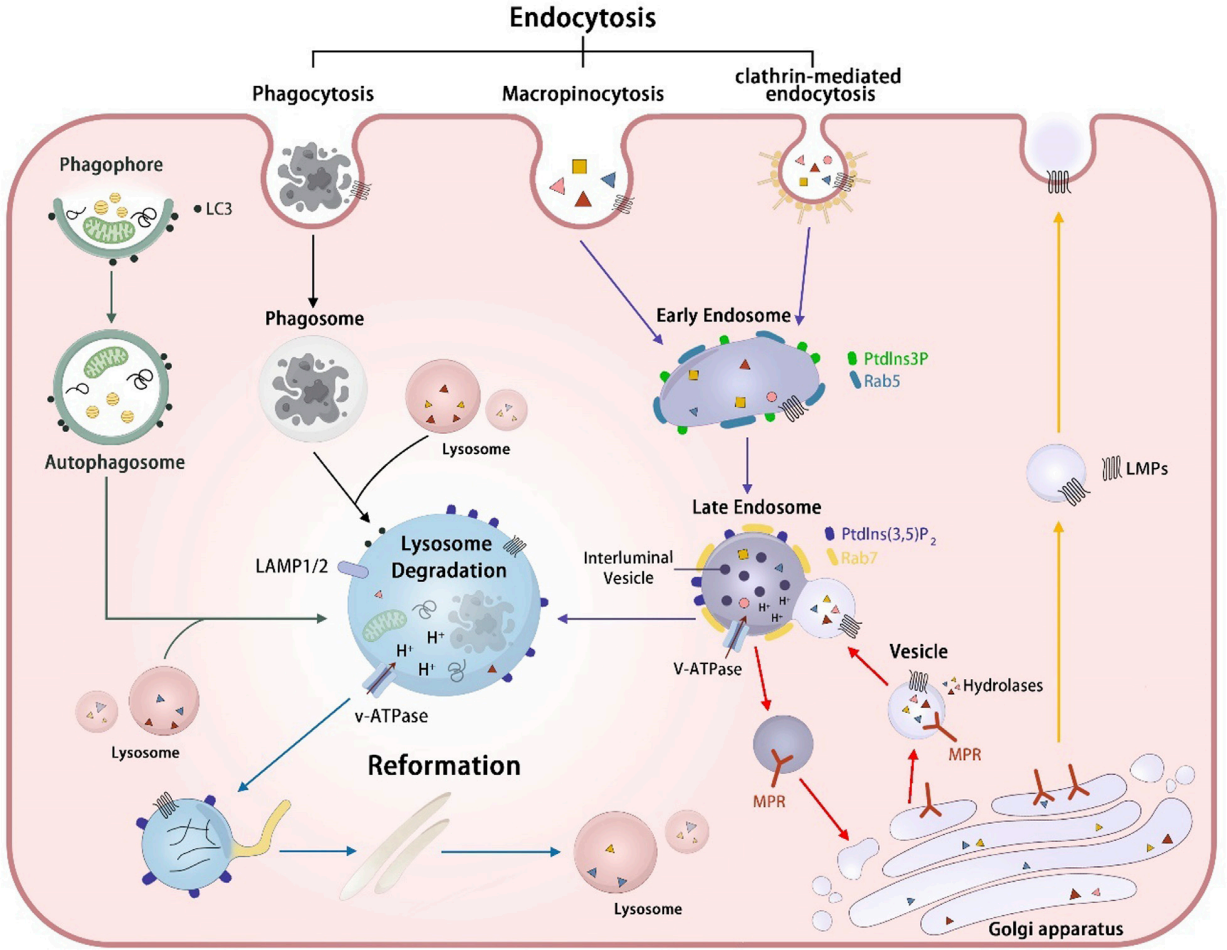
The biogenesis and degradation process of lysosomes. Lysosomal hydrolases MPR-independent targeting pathway (red arrows): Lysosomal hydrolases were delivered to the Golgi apparatus and subsequently recognized by mannose-6-phosphate receptors (MPRs). The resulting hydrolase-MPR complexes were then transported to fuse with early endosomes, ultimately forming the late endosome. After the fusion event between the vesicle containing hydrolases-MPR complexes and the early endosome, the MPRs disassociate from the hydrolases and undergo recycling back to the Golgi apparatus. Lysosome membrane protein transportation pathway (yellow arrows): Lysosomal membrane proteins (LMPs) are sorted at the Golgi apparatus and transported to endosomes directly or first transported to the cell plasma before being transferred to early endosomes through various endocytic mechanisms, including phagocytosis, micropinocytosis and clathrin-mediated endocytosis. Endosome-lysosome pathway (purple arrows) The early endosomes transform into late endosomes through fusion with vesicles and the luminal acidification formed by V-ATPase promotes the activity of lysosomal hydrolases, enhancing the degradation of cargos. Phagosome-lysosome pathway (black arrows): Phagosomes formed through cell phagocytosis mature and fuse with lysosomes, resulting in the eventual degradation of cargos contained within the phagosomes. Autophagosome-lysosome (green arrows):Autophagic cargos undergo degradation by lysosomes via the fusion of autophagosomes with lysosomes. Lysosome reformation (blue arrows):Lysosome reform from hybrid vesicle, including endolysosomes, phagolysosomes, and autolysosomes through tubulation, through processes of tubulation, budding off, and the formation of new mature lysosomes.

The synthesis and modification of hydrolases by oligosaccharides occurs in the endoplasmic reticulum, followed by transportation to the trans-Golgi network (TGN) where phosphorylation of mannose residues on their oligosaccharide chains occurs, allowing binding to the mannose 6-phosphate receptor (MPR). Subsequently, the hydrolase-MPR complexes are encapsulated within clathrin-coated vesicles and transported to early endosomes (Yang and Wang, 2021; Zhang et al., 2021) (Figure 1). During endosome maturation, a cascade of morphological and functional transitions occurs, culminating in the fusion with lysosomes to establish endolysosomal compartments. This fusion event leads to the formation of an acidic milieu within the endolysosomes, which is optimal for the activation and function of lysosomal hydrolases, thereby facilitating the degradation of internalized macromolecules. Oligosaccharides play a critical role in this process, as they are not merely structural components but also functional signals that dictate the enzyme’s destination. The correct modification of these oligosaccharides ensures proper enzyme targeting and functionality within the lysosome (Amaral et al., 2023). Additionally, some lysosomal hydrolases may be directed to endosomes through MPR-independent targeting mechanisms, including two targeting alternative receptors, sortilin and lysosomal integral membrane protein 2. Sortilin plays a role in sorting the lysosomal hydrolases, such as prosaposin, acid sphingomyelinase, cathepsin D and cathepsin H. Lysosomal integral membrane protein two is involved in the transport of β-glucocerebrosidase (Coutinho et al., 2012). In addition to the pathways mediated by targeting receptors, some lysosomal proteins without specific targeting signals may reach the plasma membrane through the constitutive secretory pathway and then arrive at lysosomes via endocytosis (Braulke and Bonifacino, 2009a).

#### 3.1.2 Lysosomal membrane protein

Lysosomal membrane proteins (LMPs) are predominantly found in the limiting membranes of lysosomes and serve various functions, such as acidifying the lysosomal lumen, facilitating the transport of degradation products, and mediating membrane fusion. Lysosome-associated membrane proteins 1 and 2 (LAMP1 and LAMP2) are the most prevalent membrane proteins, constituting approximately half of all lysosomal membrane proteins (Tong et al., 2022). Due to their abundance and localization within the membrane, LAMPs are considered to act as a protective barrier within the acidic environment of the lysosome (Cesar-Silva et al., 2022). However, recent studies have indicated that LAMPs play a role in various cellular processes, including phagocytosis, chaperone-mediated autophagy, macroautophagy, and cholesterol transport (Gu et al., 2021; Xu et al., 2023). Additionally, other important lysosomal membrane proteins, such as vacuolar-type ATPase(V-ATPase) and proton pumps located in late endosome membranes, are also essential for maintaining the acidic pH necessary for lysosomal functions (Nakanishi-Matsui and Matsumoto, 2022).

These LMPs are also derived from the TGN and are transported to lysosomes via both direct and indirect routes. The majority of LMPs are delivered to the plasma membrane via the secretory pathway and subsequently proceed to lysosomes through the endocytic pathway, whereas some LMPs are directly transported from TGN to the endolysosomal pathway (Luzio et al., 2014; Braulke and Bonifacino, 2009b) (Figure 1).

### 3.2 Lysosomal fusion and reformation

The intricate process of lysosome biogenesis involves a well-orchestrated sequence of events, including the synthesis of lysosomal proteins and endosome-lysosome fusion (Figure 1). Following post-translational modifications and sorting, lysosomal proteins are transported into the cytoplasm through clathrin-coated vesicles originating from the TGN or plasma membrane. The specifics of lysosomal protein processing and sorting have been elucidated in previous part 2.1.1 and 2.1.2. Here, we summarize the mechanisms involved in lysosomal fusion and reformation from the perspectives of both the endosomal-lysosomal system and the autophagy-lysosome system.

#### 3.2.1 Endosomal-lysosomal system

Endosomes are a type of heterogeneous vesicles formed by the endocytosis or phagocytosis of cells. Early endosomes are formed through the process of plasma membrane invagination and undergo maturation into late endosomes through continuous membrane input and output as well as material exchange. This maturation process is pivotal in the endocytic pathway, involving the replacement of key regulators specific to early endosomes. Specifically, Rab5, a small GTPase integral to the function and dynamics of early endosomes, is replaced by Rab7, a counterpart that characterizes late endosomes. Concurrently, phosphatidylinositol 3-phosphate (PtdIns3P), a phosphoinositide lipid critical for signaling and membrane trafficking at the early endosome stage, is substituted by phosphatidylinositol 3, 5-bisphosphate [PtdIns(3,5) P2]. This lipid serves as a molecular flag of late endosomes, marking the transition and functional shift from early to late endocytic compartments. These replacements underscore the intricate regulation of endosome maturation. (Striepen and Voeltz, 2023; Schink et al., 2016). Late endosomes further interact with vesicles containing hydrolases-MPR complexes and receive the hydrolases bound to MPRs in vesicles from the TGN. Within the acidic confines of the late endosome lumen, hydrolases detach from MPRs and persist within the endosomal lumen, whereas MPRs recycle back to the TGN. Subsequently, the lysosomal hydrolases degrade endocytosed macromolecular cargos (Huotari and Helenius, 2011; Scott et al., 2014; Jeger, 2020).

Lysosomes play a critical role in receiving and degrading cargoes produced through endocytosis (Yang and Wang, 2021). The rapid consumption of the lysosome pool during the degradation process underscores the importance of lysosomal regeneration for maintaining lysosomal homeostasis. Lysosomal reformation, characterized by the emergence of tubules from hybrid vesicles that subsequently bud off to form new mature lysosomes, represents a prominent pathway for replenishing the lysosome pool (Dai et al., 2019; Nanayakkara et al., 2023). As revealed via mechanistic studies, these processes are regulated by various factors, such as mechanistic target of rapamycin complex 1 (mTORC1) (Li et al., 2021), PtdIns (3, 5) P_2_ produced by PtdIns3P 5-kinase PIKfyve (Dong et al., 2010), and the lysosomal Calcium ions (Ca^2+^) channel TRPML1 (Huang et al., 2024), which regulate the release of Ca^2+^.

#### 3.2.2 Autophagosome-lysosome system

The phagophore are nascent, double-membrane structures that initiate the process of autophagy by expanding around cytoplasmic constituents, including damaged organelles or misfolded proteins, to form autophagosomes, which are critical for the sequestration and subsequent degradation of cellular components within lysosomes. Subsequently, autophagosomes fuse with lysosomes to form autolysosomes, within which lysosomal enzymes degrade damaged organelles or proteins. The fusion between autophagosomes and lysosomes is a pivotal event in the autophagosome-lysosome system, which is modulated by various factors, such as SNAREs (Pellegrini et al., 2023), small GTPases (Roy and Roux, 2020), tethering factors (Mei et al., 2023).

The process of autophagic lysosome reformation (ALR) is necessary to regenerate the lysosomes from autolysosomes, which is important to restore the lysosomal pool and maintain lysosomal homeostasis. During the ALR, phosphatidylinositol 4, 5-bisphosphate (PtdIns(4,5)P2) plays an crucial role. Recent studies have found that the dysregulation of PtdIns(4,5)P2 inhibits the ALR, consequently resulting in the lysosomal depletion and the suppression of autophagy (McGrath et al., 2021). PtdIns(4,5)P2 is predominantly found on autophagic lysosomes, where its enrichment can recruit adaptor protein 2 (Yang and Wang, 2021; Nanayakkara et al., 2023). Adaptor protein 2, in turn, facilitates the recruitment of clathrin, which plays a key role in mediating the budding of autophagic lysosomes, thereby promoting the formation of reformation tubules, a hallmark feature of ALR. The emergence of these tubules is the most prominent feature of ALR. Subsequently, the tip of the tubule sprouts to form a new prolysosome, which is transformed into a mature lysosome after the maturation stage (Chen and Yu, 2013).

### 3.3 Lysosomal consumption

The maintenance of lysosome pool homeostasis is essential for preserving cellular functionality. Recent research indicates that the abundance of lysosome pools may diminish during cell division and become depleted for degradation of autophagic and endocytic cargo, leading to a disruption in lysosome homeostasis (Yin et al., 2020). The mechanisms involved in lysosomal consumption primarily includes: 1) lysosomal exocytosis; 2) lysosomal fusion with autophagic or endocytic vesicles and consumed for cargo degradation; 3) autophagic processes.

### 3.4 The regulation of lysosome biogenesis

**FIGURE 2:**
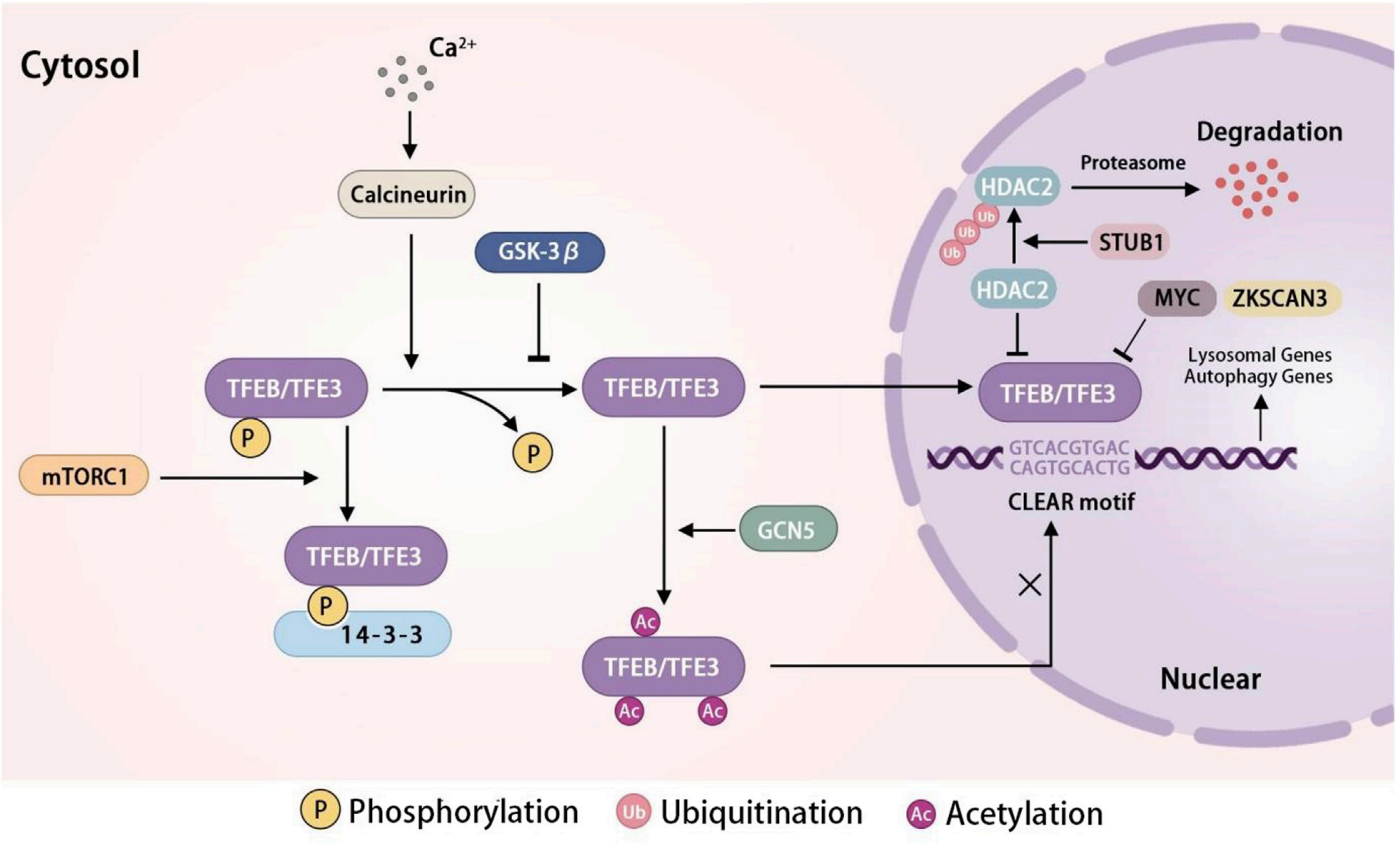
The regulation network of TFEB/TFE3 in lysosome biogenesis TFEB/TFE3 is an important transcription factor in lysosome biogenesis. In conditions of sufficient nutrients, mTORC1 phosphorylates TFEB/TFE3, resulting in TFEB binding to 14-3-3 and retention in the cytoplasm. Nutrient deprivation or cellular stress inhibits the activity of mTORC1, leading to the dephosphorylation and nuclear translocation of TFEB/TFE3. GSK-3β suppress the activity of TFEB/TFE3 by dephosphorylating it. STUB1 activates the TFEB through the ubiquitination and degradation of HDAC2. Ca^2+^ release activates the TFEB/TFE3 via calcineurin, which dephosphorylates TFEB, leading to its nuclear translocation. GCN5 inhibits the transcriptional activity of TFEB by acetylating specific lysine residues. MYC and ZKSCAN3 inhibit the transcription of TFEB. In the nuclear, TFEB/TFE3 bind with CLEAR motif to promote the expression of lysosomal genes and autophagy genes. Abbreviations: TFEB, transcription factor EB; TFE3, transcription factor E3; mTORC1, mechanistic target of rapamycin complex 1; GSK-3β, glycogen synthase kinase three beta; Ca2+, calcium ions; STUB1, STIP1 homology and U-box-containing protein 1; GCN5, general Control Non-repressed five protein; ZKSCAN3, Zinc finger protein with KRAB and SCAN domains 3; CLEAR, coordinated Lysosomal Expression and Regulation element.

Lysosomes enhance their quantity through the upregulation of lysosomal and autophagy genes at the transcriptional level in order to uphold intracellular lysosome pools’ equilibrium. Various transcription factors, specifically transcription factor EB (TFEB) and transcription factor E3 (TFE3) from the microphthalmia family (MiT/TFE), play a crucial role in orchestrating lysosome biogenesis and autophagy. Recent research has shown that TFEB and TFE3 govern the expression of numerous autophagy-related and lysosomal proteins (Settembre et al., 2013). The transcriptional factors TFEB and TFE3 upregulate lysosomal biogenesis and autophagy through interaction with the Coordinated Lysosomal Expression and Regulation element (CLEAR), a palindromic ten-base pair motif ‘GTCACGTGAC’ situated in the premotor region of lysosomal genes (La Spina et al., 2021). The activity of TFEB and TFE3 is modulated by their translocation between the cytosol and the nucleus. In conditions of sufficient nutrients, mTORC1 becomes activated and phosphorylates TFEB at multiple serine residues (S211, S142, S122) and TFE3 at serine 321 (Paquette et al., 2021). This phosphorylation promotes the binding of TFEB and TFE3 to 14-3-3 proteins, inhibiting their translocation from the cytosol to the nucleus. Conversely, during periods of nutrient deprivation or cellular stress, TFEB and TFE3 are dephosphorylated by inactivated mTORC1 and subsequently translocated to the nucleus where they stimulate lysosomal biogenesis and autophagy (Dang and Back, 2021; Zhitomirsky et al., 2018). The dephosphorylation of TFEB and TFE3 can be modulated by glycogen synthase kinase three beta (GSK-3β) through the protein kinase C and the eukaryotic translation initiation factor 4A-3 signaling pathways (Pan and Valapala, 2022). Calcineurin is activated by Ca^2+^ released from lysosomes via TRPML1 in reaction to various lysosomal stressors, leading to the translocation of dephosphorylated TFEB to the nucleus and enhancing the transcription of autophagic and lysosomal genes (Hu et al., 2023). MYC, the transcription factor characterized by its basic helix-loop-helix structure, can bind to the promoters of genes targeted by TFEB, inhibiting the binding and activation of TFEB for transcription (Wu and Eisenman, 2021). ZKSCAN3, a zinc-finger protein containing KRAB and SCAN domains, serves as a key transcriptional repressor of genes involved in lysosomal and autophagy processes that are targeted by TFEB (Ouyang et al., 2023; New-Aaron et al., 2021; Ding et al., 2022). In addition to phosphorylation, TFEB is subject to regulation through glycosylation, cysteine oxidation, and acetylation. The post-translational modification of TFEB through the acetylated lysines has been identified as a mechanism for regulating its activity (Franco-Juárez et al., 2022). Recent research had shown that General Control Non-repressed five protein, the histone acetyltransferase disrupted the dimerization and transcriptional activity of TFEB by acetylating specific lysine residues (K116, K274, and K279), ultimately inhibiting lysosomal biogenesis (Wang Y. et al., 2020). STIP1 homology and U-box-containing protein 1 has been shown to facilitate the activation and nuclear translocation of TFEB through the ubiquitination and degradation of HDAC2 (Lu et al., 2023). Consequently, the biosynthesis of lysosomes is regulated by the activation of TFEB or TFE3 in the cytoplasm and their subsequent transcriptional activity in the nucleus (Figure 2).

Apart from the major regulator TFEB, several other regulators for lysosome biogenesis have been reported as well. BRD4 has been recognized as a potential suppressor of autophagy, capable of inhibiting the transcriptional activity of lysosomal genes through its interaction with the Histone H4 lysine 16 residue (H4K16) at the promoters of these genes (Gong et al., 2022). Moreover, signal transducer and activator of transcription 3(STAT3) has been implicated in the regulation of lysosomal gene expression. Lysosomal protease deficiency or protein overload leads to the generation of lysosomal reactive oxygen species and activation of STAT3, which in turn can stimulate the transcription of key lysosomal proteolytic hydrolases. Recent research has indicated that STAT3 may additionally interact with V-ATPase and enhance its function, indicating that the role of STAT3 in regulating lysosomal acidification is independent of its transcriptional capabilities (Liu et al., 2018).

## 4 Lysosomes modulate the OCs differentiation

Lysosomes serve as conventional storage organelles for intracellular Ca^2+^, a crucial signaling messenger in cells. Fluctuations in cytoplasmic Ca^2+^ levels can activate various pathways and impact cellular functions such as proliferation, differentiation, migration, and apoptosis (J et al., 2022). OCs, the specific bone-resorbing cells, are integral in maintaining bone homeostasis. Recent research has highlighted the essential role of Ca^2+^ in the regulation of OC differentiation. The knockdown of P2X7R, an ion channel receptor, might decrease the Ca^2+^ influx and impacts the intracellular Ca^2+^ levels, which lead to the repression of the nuclear factor of activated T cells 1 (NFATc1)- mediated gene transcription, thereby inhibiting the differentiation of OCs (Ma et al., 2022). Additionally, transient receptor potential mucolipin 1, two-pore channel, and two-transmembrane channel for trimeric Ca^2+^ (P2X4) are three major ion channels located in the lysosomal membrane that regulate the release of lysosomal Ca^2+^. The knockdown and depletion of TRPML1 can prevent the release of lysosomal Ca^2+^ and block the process of Ca^2+^ oscillations, thereby impeding the activation of NFATc1 and osteoclastogenesis genes (Erkhembaatar et al., 2017). Two-pore channel 2 has been identified as crucial for OC differentiation, as evidenced by the hindrance of RANKL-regulated processes, including NFATc1 activation and Ca^2+^ release, in two-pore channel 2-knockdown cells (Webb et al., 2020). In addition to the Ca^2+^ signal, the process of lysosome biogenesis also affects the differentiation of OCs. Rab11A and Rab34, members of the small GTPase family, facilitate the proteolysis of osteoclastogenic receptors c-fms and RANK through the early endosomes–late endosomes–lysosomes pathway, leading to the suppression of transcriptional activity of c-fos, NFATc-1, and OC differentiation (Okusha et al., 2020; Feng et al., 2022). Tet methylcytosine dioxygenase 2 (TET2) functions as a DNA demethylase by oxidizing 5-methylcytosine to 5-hydroxymethylcytosine. Knockdown of TET2 also results in decreased formation of autophagic vesicles through upregulation of BCL2 expression (Yang et al., 2022). Rab interacting lysosomal protein (RILP) could reduce the migration of preosteoclasts by PI3K/AKT signaling to regulate the formation of OCs (Wu et al., 2023). It can be concluded that lysosomes play a crucial role in OC differentiation, with lysosomal Ca^2+^ and proteins serving as important regulators. Due to the complexity of OC differentiation and the involvement of multiple factors, it is probable that additional lysosomal regulators or pathways are implicated in this process and warrant further investigation.

## 5 Lysosomes in bone resorption of OCs

### 5.1 Overview of bone resorption

**FIGURE 3:**
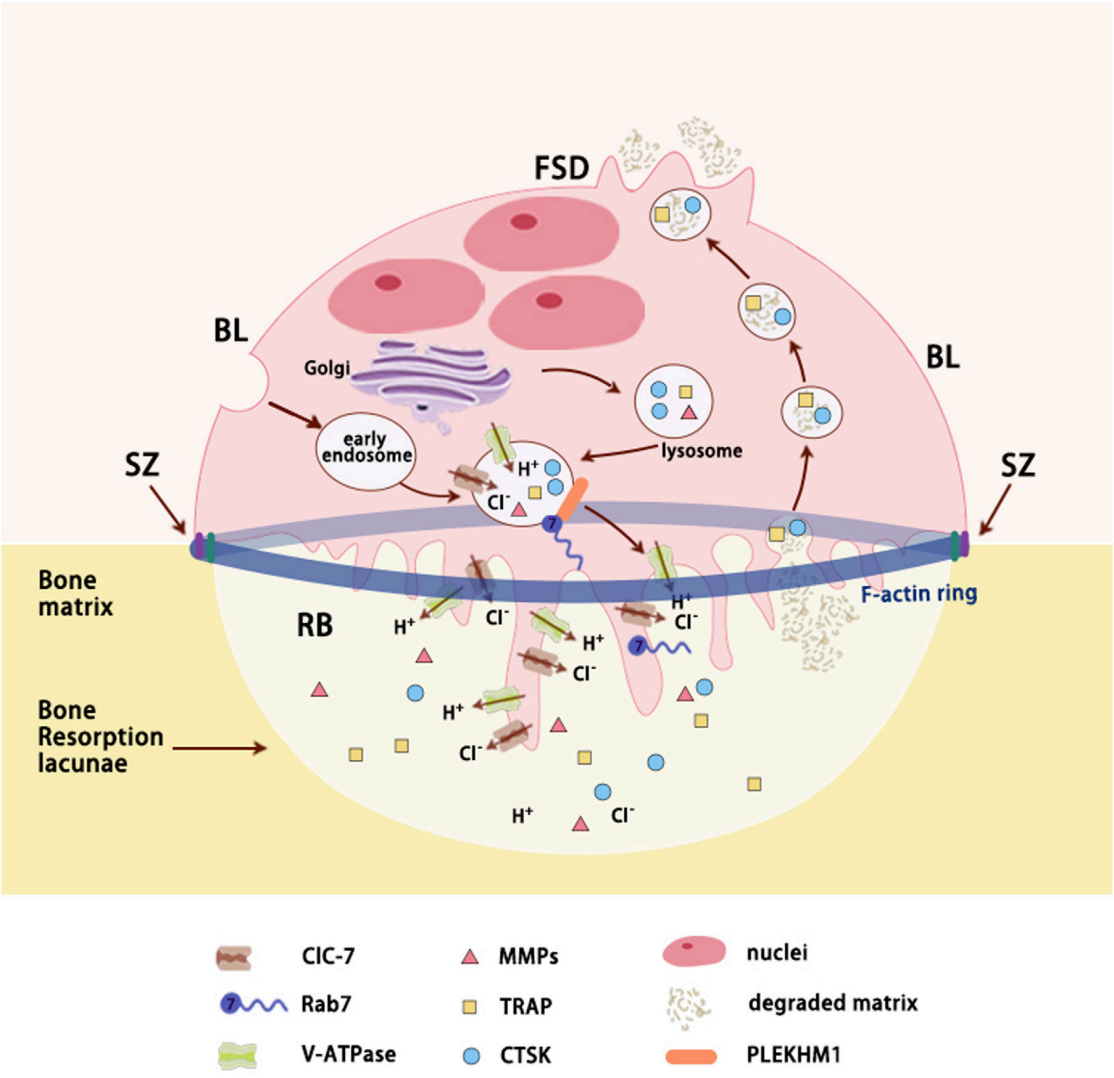
Secretory lysosomes trafficking in resorbing OCs. Polarized osteoclasts (OCs) adhere to bone matrix by a sealing zone composed of actin ring that acts like a gasket encompassing the ruffled border. Secretory lysosomes derived from endosomal pathway transport lysosomal proteins to ruffled border via Rab7 and its downstream effector (such as PLEKHM1). As Lacunae acidification proteins inserted into ruffled border (RB) and proteolytic enzymes released in bone resorption lacunae, bone matrix is degraded and then is reuptook by RB through transcytosis. TRAP and CTSK, which are localized within transcytotic vesicles, are involved in the reprocessing of the degraded matrix. Ultimately, degradation products are released at the FSD. Abbreviations: RB, ruffled border; SZ, sealing zone; FSD, functional secretory domain; BL, basolateral membrane; CLC-7, chloride channel type 7; V-ATPase - Vacuolar-type ATPase; MMPs, matrix metalloproteinases; TRAP, tartrate-resistant acid phosphatase; CTSK, cathepsin K; PLEKHM1, protein family member 1.

OCs, large multinucleated cells derived from hematopoietic precursors, possess secretory lysosomes with distinctive regulated exocytic ability, similar to other cell types such as melanocytes and cytotoxic lymphocytes (Gros and Muller, 2023; Kodama and Kaito, 2020). The process of bone resorption involves a dynamic multi-step cycle that commences with the polarization of OCs and the reorganization of their cytoskeleton (Figure 3). The plasma membrane of activated OCs can be subdivided into four distinct domains: ruffled border (RB), sealing zone (SZ), functional secretory domain (FSD), and basolateral membrane (BL) (Hou et al., 2023). Subsequently, activated OCs form SZ rich in filamentous actin (known as actin ring) via αvβ3 integrins in order to attach to the bone surface and create isolated resorption lacunae. Following tight adhesion to the bone matrix, the plasma membrane facing the bone fuses with late endosomal vesicles to form the RB, significantly increasing surface area (Hou et al., 2023; Soysa and Alles, 2016; Ng et al., 2019). The RB serves as a specialized resorbing organelle that releases protons and lysosomal proteases through vesicular cytosis to the isolated resorption compartment. Therefore, the RB combined with resorptive lacunae is referred to as a “giant extracellular lysosome” (Väänänen et al., 1990). The maintenance of RB is implicated with the continuous retrieval of plasma membrane and fusion of biosynthetic and transcytotic vesicular trafficking (Na et al., 2020). Interestingly, proteins related to autophagy, including Atg7, Atg4B, and LC3, are suggested to play a role in RB development (Soysa and Alles, 2016).

As bone matrix is the complex of both organic (mostly type I collagen) and inorganic (mainly crystalline hydroxyapatite ([Ca_3_(PO_4_)_2_]_3_Ca(OH)_2_) components, resorptive lacunae requires acidic microenvironment to dissolve mineral as well as proteolytic enzymes to degrade organic matrix (Yang and Wang, 2021; Gong et al., 1964). The acidification of the lacunae in bone resorption is reliant upon the coordinated action of various lysosomal proteins, including active macromolecule membrane transporters (primarily V-ATPase), ion channels (such as ClC-7), and lysosomal membrane proteins (such as OSTM1), collectively referred to as acidification proteins (Lacombe et al., 2013; Ng et al., 2019; Xu et al., 2007). This acidic microenvironment serves a dual purpose in bone resorption, facilitating both the activation of proteolytic enzymes and the sealing of resorptive pits, as supported by existing evidence. The degraded bone matrix, including collagen fragments, calcium, and phosphate, is primarily transported by RB through transcytosis and released at the FSD, the membrane opposite to the RB (Veis and O Brien, 2023). Molecules smaller than 100 kDa may passively infiltrate through the SZ, but their contribution is minimal (Coxon and Taylor, 2008) (Figure 3).

### 5.2 Lysosomal protein and bone resorption

Lysosomal proteins for bone resorption can be classified into two primary categories: lacunae acidification proteins for dissolving mineral within acidic lacunae and proteolytic enzymes for degrading organic matrix (see Table 1).

**TABLE 1:** The functions of known lysosomal proteins (lacunae acidification protein and proteolytic enzymes) involved in bone resorption.

Protein	Function	Category	Citation
V-ATPase	Maintenance of acidic microenvironment in lysosomal and resorptive lacunae; Location of Rab7 and Rab27a	Lacunae acidfication protein	(Matsumoto et al., 2018; Matsumoto and Nakanishi-Matsui, 2019; Zhao et al., 2001)
ClC-7	Maintenance of ionic homeostasis in lysosomal and resorptive lacunae; Presumable involvement with vesicular trafficking		(Saito et al., 2007; Weinert et al., 2010; Graves et al., 2008)
Ostm1	Prevention of ClC-7 from proteolysis as β subunit of ClC-7		(Schrecker et al., 2020; Zhang et al., 2020a; Lange et al., 2006)
ClC-3	Involvement in acidifying lysosomal and resorptive lacunae		Okamoto et al. (2008)
CTSK	Degradation of type I collagen and several non-collagen proteins; Involvement in lysosomal proteins transport	Proteolytic enzymes	(Ghosh et al., 2003; Brömme and Okamoto, 1995; Rosen et al., 1994)
MMP-9	Degradation of organic matrix via interaction with MMP-14		Zaidi et al. (2001)
MMP-14	Degradation of organic matrix via interaction with MMP-9		Zaidi et al. (2001)
MMP-13	Substitute for bone collagenolysis in pathological condition		Delaissé et al. (2003)
TRAP	Further processing of degraded collagen		(Vääräniemi et al., 2004; Yamaza et al., 1998; Halleen et al., 1999)

#### 5.2.1 Lacunae acidification protein

The V-ATPase, initially identified in yeast and plant vacuoles, is a widely distributed macromolecular complex that transports protons using energy derived from ATP hydrolysis in mammals (Futai et al., 2019). It is functionally divided into two sectors: the catalytic V_1_ sector and the proton-translocating V_0_ sector. The peripheral V_1_ sector, consisting of eight subunits (A_3_, B_3_, C_1_, D_1_, E_3_, F_1_, G_3_, and H_1_), generates energy through ATP hydrolysis and initiates the rotation of the V_0_ sector. In contrast, the membrane-bound V_0_ sector, composed of six subunits (a, d, e, c, c', and c''), is energized by the V1 sector and facilitates the movement of protons across biological membranes (Vasanthakumar and Rubinstein, 2020; Ribet et al., 2021). In response to the acidifying resorption area, OCs have evolved specific subunit isoforms, a3 and d2, which are expressed in secretory lysosomes. The upregulation of these expressed isoforms during differentiation suggests that the V-ATPase equipped with them serves as the primary proton pump in OCs (Nakanishi-Matsui and Matsumoto, 2022; J et al., 2003; H et al., 2009). Additionally, a high-affinity interaction between the a3 and d2 isoforms is demonstrated by GST pull down assay. The a3 isoform is genetically encoded by *TCIRG1* in humans and *ATP6i* in mice. The knock out of a3 gene caused a phenotype of osteopretrosis (abnormally high bone density) owing to the inability to acidify lacunae in OCs, consistent with the phenotype observed in all-type mutations of the a3 gene (Nakanishi-Matsui and Matsumoto, 2022; Ribet et al., 2021; Matsumoto et al., 2018). Conversely, deficiencies in the H or G1 isoforms led to osteoporosis (decreased bone density) in mice, highlighting the multiple role V-ATPase isoforms play in the bone homeostasis (Duan et al., 2018). In the process of bone resorption, V-ATPase in secretory lysosome was transported and inserted into RB with the aid of a3 isoform to provide a large amount of acid equivalents necessary to dissolve mineral (Veis and O Brien, 2023). Likewise, V-ATPase Ac45 was found to regulate OC acidification and bone resorption (Feng et al., 2008; Qin et al., 2011). Consistently, suppression of V-ATPase by small molecule inhibitors such as balfilomycin and saliphenylhalamide lead to the inhibition of OC acidification and function (Qin et al., 2012; Zhu et al., 2016a).

In order to regulate the ionic balance within lysosomes and resorption lacunae in OCs, the transportation of Cl^−^ ions is primarily facilitated by chloride channel type 7(ClC-7) (Zhao et al., 2009a). As a widely distributed member of the CLC chloride channel family, ClC-7 functions as a CL^−^/H^+^ antiporter situated in late endosomes, lysosomes, and the RB of bone-resorbing OCs (Stauber et al., 2012). Studies on OCs attached to ivory in CLC-7 knockout mice have demonstrated an inability to acidify resorption lacunae, as indicated by staining with the pH-sensitive dye acridine orange (Kornak et al., 2001). However, conflicting research suggested that mutations in *CLC7N*, the gene encoding ClC-7, did not impact the pH levels within the lumen. The Cl^−^ flux is proposed to play a pivotal role in vesicular trafficking within early endosomes by modulating the influx of lysosomal Ca^2+^ (Sobacchi et al., 2013). In contrast to other ClC chloride channels, ClC-7 requires the distinct β-subunit osteoclastogenesis associated transmembrane protein 1 (Ostm1) for its specialized functions in bone resorption and lysosomal activity. Recent studies utilizing cryoelectron microscopy have elucidated the mechanism underlying the interaction between ClC-7 and Ostm1. The intravesicular domain and transmembrane domain of ClC-7 are shielded by the highly glycosylated Ostm1 subunit, providing protection against proteolysis within lysosomes and the resorptive region (Schrecker et al., 2020; Zhang S. et al., 2020). ClC-3 has been identified on late endosomes and is implicated in bone resorption through acidification of the lumen (Okamoto et al., 2008). Resorbing OCs also express ClC-4, ClC-5, and ClC-6, although the specific functions and subcellular locations of these chloride channels remain ill-defined (Stauber et al., 2012).

#### 5.2.2 Proteolytic enzymes

The proteolytic resorption of exposed organic matrix occurs after the dissolution of the inorganic matrix, as the activation of proteolytic enzymes depends on acidic mircroenvironment. Among them, cathepsin K(CTSK), a cysteine protease belonging to the papain family, is predominantly expressed in OCs. As is mentioned before, over 90% of the organic composition of bone consists of type I collagen, with the remaining portion comprising non-collagenous proteins such as osteocalcin, osteonectin, osteopontin, fibronectin, thrombospondin, and bone sialoprotein. Type I collagen composes covalently cross-linked triple helices formed by two α1I) and one α2I) chains (Lecaille et al., 2008). Unique among cysteine protease, CTSK is able to degrade both helical and telopeptide domains of type I collagen as well as the osteonectin and osteopontin (Zaidi et al., 2001; Costa et al., 2011). As high exclusive expression level of mature CTSK is demonstrated in OCs near bone surface, it can be speculated that immature CTSK is activated in lysosomal luminal with a potential regulator, phosphatidylinositol 3-kinases, and then secreted to resorption area as mature CTSK (Costa et al., 2011). In addition to OCs, CTSK is expressed in various tissues, including bone, ovary, heart, placenta, lung, skeletal muscle, colon, and small intestine, albeit at low levels (Zhao et al., 2009b). Mutations in CTSK can lead to pycnodysostosis, a rare autosomal recessive skeletal dysplasia syndrome (Mijanović et al., 2022). In CTSK knockout mice, OCs exhibit impaired degradation of the organic bone matrix, while biogenesis and demineralization remain unaffected (Costa et al., 2011). Increased OC numbers in some CTSK knockout mice may be attributed to the involvement of CTSK in apoptosis. Furthermore, CTSK contains a mannose-6-phosphate moiety that interacts with MPRs to facilitate transport of lysosomal proteins to endo-lysosomal system (Ghosh et al., 2003).

In addition to CTSK, matrix metalloproteinase (MMP) also contribute to the solubilization of organic components. Members of the MMP family possess the ability to cleave type I collagen at specific peptide bonds within the triple-helical domain (Yasuda et al., 2005). However, the enzymatic activity of MMPs extends beyond bone resorption, which means they are also involved in OC differentiation, OC migration and so on. Certain MMPs found in resorption lacunae, such as gelatinases MMP-2 and MMP-9, and collagenases MMP-13 and MMP-14, have been identified as participants in collagenolysis (Delaissé et al., 2003). They seem to engage in a compensatory network in bone collagenolysis, the mechanism of which remains unclear, as the deficiency of each of these enzymes did not lead to a bone phenotype. MMP-9, primarily expressed in OCs, was suggested to potentially work in tandem with MMP-14 based on the osteopetrotic phenotype observed and the reduced bone resorbing activity seen in Mmp-9/Mmp-14 double knockout mice. Moreover, the concurrent inhibition of MMP-9 and MMP-14 has been shown to reduce bone loss in mice with parathyroid hormone or ovariectomy, suggesting a promising therapeutic approach for osteoporosis (Zhu et al., 2020). In pathological conditions such as CTSK deficiency or tumor-induced osteolysis, non-osteoclastic MMP-13 may potentially compensate for collagen degradation in bone, as evidenced by the consistent rate of collagen breakdown independent of MMP-13 (Delaissé et al., 2003). Additional research is necessary to draw a definitive conclusion.

Tartrate-resistant acid phosphatase (TRAP), commonly utilized as a biomarker for OCs, has been found to co-localize with CTSK and collagen fragments within transcytotic vesicles. It has been observed that the phosphatase activity of TRAP can be enhanced by CTSK, suggesting a potential role for TRAP in the further degradation of collagen, a process that may also involve reactive oxygen species generated through its redox-sensitive iron atom. The presence of a neutral pH environment within transcytotic vesicles indicates that ROS, which can adapt to this pH level, may have a more significant impact compared to the phosphatase function of TRAP (Coxon and Taylor, 2008; Zhao, 2012; Vääräniemi et al., 2004).

### 5.3 The regulation of lysosomes in bone resorption

OCs have two different lysosomal systems: conventional lysosomes, which primarily contain acidic hydrolase cathepsin D and are responsible for maintaining intracellular homeostasis, and secretory lysosomes, which play a crucial role in bone resorption by promoting the formation of RB, releasing lysosomal acid hydrolases, and acidifying the resorptive microenvironment (H, 2012). During the bone resorption, the biogenesis, trafficking and fusion with plasma of secretory lysosomes is considered the crucial phase for various regulators (see Table 2) to modulate bone resorption activity.

**TABLE 2:** OC proteins involved in regulating secretory lysosomes during bone resorption.

Protein	Location	Function	OC function	Citation
Gasdermin D	Plasma membrane, cytosol, early endosome	Regulation of pyroptosis	Lysosomal maturation and secretion	Vääräniemi et al. (2004)
GlcNAc-1-phosphotransferase	Golgi apparatus	Formation of the mannose-6-phosphate, targeting lysosomal proteins to the endo-lysosomal pathway	Secretory lysosome biogenesis	Zhu et al. (2020)
RANK	Plasma membrane	Formation of transmembrane complex by binding with RANKL, delivering an effective stimulus		Zhao (2012)
Rab7	Late endosomes, lysosomes, RB	Regulation of fusion between early and late endosomes and between late endosomes and lysosomes; Transport of secretory lysosomes to RB	Secretory lysosome trafficking	(Matsumoto et al., 2018; Palokangas et al., 1997; Feng et al., 1995)
Rac1	Cytosol, RB	Interaction with Rab7; Regulation of the actin cytoskeleton		(Margiotta and Bucci, 2019; Sun et al., 2005)
PLEKHM1	Late endosomes, lysosomes	Interaction with Rab7; Regulation of the terminal stages of endocytic and autophagy pathways		(Fujiwara et al., 2016; McEwan et al., 2015; Baba et al., 2019)
RILP	–	Regulation of the Cathepsin K directly/indirectly secretion		Wu et al. (2023)
Rab27a	Late endosomes, lysosomes	Regulation of the plasma membrane and lysosomal membrane fusion		(Baba et al., 2019; Shimada-Sugawara et al., 2015)
Syt VII	Lysosomes	Regulation of membrane fusion by interaction with syntaxin 4 (?)		Wang et al. (2005)
Rab3D	Post-TGN vesicles	Regulation of TGN trafficking to RB; Involvement in calcium delivery by interacting with Calmodulin		(Pavlos et al., 2005; Zhu et al., 2016b; Abu-Amer et al., 1999)
Tctex-1	Rab3D-bearing vesicles	Interaction with Rab3D as a molecular adaptor of dynein motor complex		Pavlos et al. (2011)
Rab9	Endosomes, lysosomes	Involvement in cargo sorting and vesicular budding from endosomes		Zhao et al. (2002)
Rab13	Vesicles between TGN and basal plasma membrane	Regulation of secretory lysosomes transport via interaction with endospanin-2		(Hirvonen et al., 2012; Hirvonen et al., 2013)
SNX10	Endosomes, lysosomes	Involvement in post-TGN pathway and/or localization of V-ATPase (?); Associated with the activation and transportation of MMP9		(Chen et al., 2012; Sultana et al., 2020; Zhou et al., 2017)

#### 5.3.1 Secretory lysosomes biogenesis

The proteins in secretory lysosomes, including MMP, CTSK, and TRAP, are synthesized in ribosomes and modified in the endoplasmic reticulum, then transported to TGN. Some proteins, such as CTSK and TRAP, were modified with phosphomannosyl residues which is specifically bound with MPR, and then is transported from TGN to endocytic compartments, where the acid environment results in the disassembly of the enzymes-MPR complexes. The released enzymes are packed into the secretory lysosomes, whereas MPRs recycle back to the TGN. Other enzymes, such as CTSK, have been reported that their sorting process is MPR-independent pathway (Amaral et al., 2023).

Secretory lysosomes biogenesis process, including the synthesis and sorting of protein enzymes, endosome-lysosome fusion, is regulated by various factors. Research has shown that the sorting of CTSK and TRAP from TGN to secretory lysosomes was suppressed due to the distribution of MPR targeting pathway, leading to decreased formation of secretory lysosomes and inhibition of bone resorption activity (Vääräniemi et al., 2004). The knockdown of GlcNAc-1-phosphotransferase leads to disruption of the MPR pathway, thereby inhibiting the formation of the secretory lysosome (Zhu et al., 2020). Moreover, it has been demonstrated that gasdermin D (GSDMD) limited the formation and function of secretory lysosomes through the endo-lysosomal pathway. The depletion of GSDMD promoted the lysosomal intracellular activity and bone-resorption activity (Li et al., 2022). RANKL play an important role in regulating the biogenesis of secretory lysosomes in OCs, among which protein kinase Cβ mediates RANKL-induced secretory lysosomal biogenesis via the phosphorylation of TFEB on three serine residues, S462, S466, and S468 (M et al., 2013).

#### 5.3.2 Secretory lysosome transport and fusion with plasma

During the process of bone resorption, the regulation of cargo delivery and plasma membrane recycling through vesicular trafficking necessitates the involvement of various regulators, including the Rab GTPase family and SNX proteins. The Rab GTPase family, which comprises approximately 70 members in mammals, can be classified into two groups based on their molecular weights: small Rab GTPases (20–30 kDa) and large Rab GTPases (70–150 kDa) (Tsukuba et al., 2021; Y et al., 2021). These GTPases serve as key coordinators of membrane transport, existing in two reversible states: an active GTP-bound state and an inactive GDP-bound state. Indeed, the GTP-bound/GDP-bound switch is regulated by guanine nucleotide exchange factors (GEFs) responsible for GTP/GDP exchange and GTPase-Activating Protein for GTP-hydrolysis. In active state, Rab proteins recruit numerous effector which interacts with actin or microtubule-related motor proteins to regulate multiple stages of vesicle trafficking ranging from budding to fusion (Roy and Roux, 2020; Lamber et al., 2019; Bhuin and Roy, 2014). The localization and function of Rab GTPase family rely on prenylation, a practical approach to enables proteins to efficiently bind to lipid bilayers, thereby facilitating their dynamic recruitment from membranes for regulatory purposes. Impaired prenylation may lead to OC apoptosis (Coxon et al., 2006). Studies have found that dual prenylation is essential to the functionality of Rab GTPase, as mono-prenylated Rab protein failed to support the growth of yeast in limited temperature and localize to correct intracellular membrane. This deficiency was likely attributed to their limited ability to effectively integrate into the lipid bilayer of the modified protein (Calero et al., 2003). Furthermore, the newly synthesized Rab protein, in its GDP-bound state, is known to be recognized by the Rab escort protein. The Rab escort protein then hands it over to the Rab geranylgeranyl transferase RGGT, an enzyme that catalyzes the geranylgeranylation of one or two cysteine residues at the carboxy terminus of the Rab proteins. The release of prenylated, inactive Rab GTPase from subcellular membranes into the cytosol is facilitated by GDP dissociation inhibitor binding to the hydrophobic groups of Rab GTPase. GEF plays a role in transporting Rab GTPase to new target membranes by releasing GDP dissociation inhibitor (Roy and Roux, 2020).

Membrane-bound transport is mainly achieved by Rab7, a small GTPase residing in late endosome/lysosome. In resorbing OCs, GDP-bound Rab7 is recruited by V-ATPase a3 isoform in secretory lysosomes, and is subsequent to be activated to be GTP-bound Rab7 presumably by Mon-Ccz1, the GEF which co-localizes with V-ATPase a3 isoform (Nakanishi-Matsui and Matsumoto, 2022; Matsumoto et al., 2022; Matsumoto and Nakanishi-Matsui, 2019). Among various downstream effector, Rac1 and protein family member 1(PLEKHM1) are two identified one recruited by GTP-dependent Rab in OCs. Rac1, a small Rho GTPase able to control actin cytoskeleton, is thought to collaborate with Rab7 to mediate RB expansion by indirectly connecting microtubules and microfilaments that allows late endosome to be transported to RB in OCs (Margiotta and Bucci, 2019; Sun et al., 2005). Comparatively, PLEKHM1 is known to mediate endocytic fusion through binding to the GTP-bound form of Rab7 in aid of DEF, and followed recruitment of FAM98A and NDEL1 (Fujiwara et al., 2016). Moreover, both the mutation and depletion of PLEKHM1 can cause phenotype of osteopetrosis with OCs deficient in RB and bone resorption *in vivo* (McEwan et al., 2015). Besides, RILP has been suggested to play a role in bone resorption function of OCs due to inhibited secretion of CTSK caused by RILP inhibitor (Wu et al., 2023). In terms of the fusion of plasma membrane with lysosomal membrane with Rab27a as a mobility driver, V-ATPase a3 isoform is involved in the localization of GDP-bound Rab27a (M et al., 2010). Rab7 is also redistributed to the domain of RB, where it co-localizes with V-ATPase and CTSK, aligning with the pathological manifestation of diminished CTSK secretion, bone resorption, and OC polarization resulting from Rab7 downregulation (Ng et al., 2019; Vaananen, 2005). Syt VII, a lysosomal-associated protein, may contribute to the membrane fusion by binding to syntaxin 4, a plasma membrane SNARE protein (Zhao, 2012; Wang et al., 2005).

In addition to Rab7 and Rab27a, there is limited information about other Rab GTPases involving in vesicular trafficking. In mature OCs, Rab 13 residing in small vesicle between TGN and basolateral membrane is likely to mediate secretory functions by interaction with endospanin-2, a small transmembrane protein (Hirvonen et al., 2012; Hirvonen et al., 2013). Rab9, which has been observed to co-localize with Rab7 in late endosomes surrounding nuclei, plays a role in sorting cargo and promoting vesicular budding from endosomes (Zhao et al., 2002). Rab3D, the most prevalent Rab3 isoform (Rab3 A/B/C/D) expressed in OCs, is thought to be involved in RB maintenance by modulating a TGN trafficking step that is distinct from conventional endocytic trafficking (Pavlos et al., 2005). Tctex-1 is identified as an interaction partner of Rab3D that bridges membrane-microtubule transport through cytoplasmic dynein (Pavlos et al., 2011). Additionally, Calmodulin has been found to interact with Rab3D at the RB, potentially aiding in the delivery and calcium sensitivity of Rab3D-containing vesicles and thereby influencing bone resorption (Zhu et al., 2016b). More recently, member a2 of the solute carrier 37 family (Slc37a2) as a SL sugar transporter was found to regulate secretory lysosomes in OC bone resorptive function (Ng et al., 2023).

Sorting nexin (SNX) family is composed of various peripheral membrane protein with a shared phospholipid-binding domain involved in modulating protein sorting and cargo trafficking. The SNX10 expression is evidently upgraded during RANKL-induced osteoclastogenesis *in vitro* and is expressed in OCs *in vivo*. Based on studies performed by shRNA-mediated depletion of SNX10, SNX10 is required for TRAP secretion and resorption function of OCs. Research conducted in zebrafish and immortalized HeLa cells indicates that SNX10 may play a role in the post-TGN trafficking process and/or the localization of V-ATPases within vesicles (Chen et al., 2012; Sultana et al., 2020; Zhou et al., 2017). Further investigations are needed to validate this hypothesis. Additionally, SNX10 has been linked to the transportation and activity of MMP9 through the JNK-p38-ERK signaling pathway (Zhou et al., 2017).

## 6 Lysosomes in autophagy of OCs

In addition to participate in the process of bone resorption, lysosomes are also involved in the autophagy of OCs. Autophagy, a well-conserved catabolic pathway in eukaryotic cells, was initially observed under conditions of nutrient stress. This process allows for the recycling of small molecule nutrients and energy, thereby supporting cellular function and metabolism (Ou et al., 2022). Recent studies have reported that autophagy is closely associated with OC differentiation, migration and function. Specifically, during the process of RANKL-induced OC differentiation, there was a notable upregulation in the expression of autophagic proteins such as ATG5, ATG7, and LC3. This increase in expression led to the formation of actin rings, which play a crucial role in regulating the initiation of osteoclastogenesis (Tong et al., 2022; Li et al., 2014). Tannic acid, a polyphenolic compound, was reported to decrease the quantity of autophagic vesicles and the expression levels of autophagic proteins such as LC3B, BECN1, ATG5, and ATG7 by deactivating AKT, ultimately leading to the inhibition of OC differentiation (D et al., 2022). In a separate study, the suppression of autophagy, specifically through LC3B depletion, led to an increase in kindlin3 levels and promoted the interaction between kindlin3 and integrin β3, resulting in the disassembly of the actin cytoskeleton and impaired migration of OCs (Zhang Y. et al., 2020). Other studies have suggested that autophagic proteins also are involved in the formation of RB and the release of tissue proteinase K, which is the key factor for maintaining normal bone resorption process (DeSelm et al., 2011).

Lysosomes play a crucial role in regulating autophagic responses in OCs. Autophagy can be categorized into three types: chaperone-mediated autophagy, micro-autophagy, and macro-autophagy, based on the mechanism by which autophagic substrates are delivered to lysosomes (Chen et al., 2023). Among these types, macro-autophagy has been the subject of extensive research and will be the primary focus of the subsequent chapters, hereafter referred to simply as autophagy.

In the process of autophagy, a membrane cistern called the phagophore (also referred to as the isolation membrane) expands around damaged organelles or misfolded proteins, enclosing them within a double-membrane structure known as an autophagosome (Mahmutefendić Lučin et al., 2022). Subsequent fusion of the autophagosome with lysosomes results in the formation of autolysosomes, where the degradation of cargo by lysosomal enzymes takes place. This fusion event between autolysosomes and lysosomes, crucial to the autophagic process, is regulated by a variety of proteins, including Beclin-1, PLEKHM1, and Rab7. Beclin-1, a crucial regulatory protein, promotes the formation of autolysosomes through the fusion of autophagosomes with lysosomes. The knockdown of Beclin-1 impaired autophagosomes fusion with lysosomes and reduced autophagy flux, thereby inhibiting the differentiation of OCs (Tong et al., 2021). It was also reported that IL-17A regulates OC autophagy via ERK/mTOR/Beclin1 signal and promotes the differentiation of OCs (Tang et al., 2023). PLEKHM1 is recognized as an essential lysosomal protein involved in the regulation of lysosome positioning and secretion. Recent studies have reported that PLEKHM1 interacts with autophagic protein LC3 to regulate autophagosome-lysosome fusion, thereby enhancing the bone resorbing activity of OCs (Fujiwara et al., 2016). Moreover, Rab7 has been shown to be involved in the autophagosome-lysosome fusion. PLEKHM1 could interact with Rab7, HOPS-SNARE complexes, and LC3 proteins to facilitate autophagosome-lysosome fusion, thereby modulating the bone resorption activity of OCs (McEwan et al., 2015). Also importantly, SQSTM1/p62 was found to regulate proteins by targeting them to the ubiquitin-proteasome system or the autophagy-lysosome pathway in OCs and bone diseases (Sultana et al., 2021; Rea et al., 2013).

## 7 Lysosomes in bone disorders

Due to its ubiquitous function in disease pathology, lysosomes as a therapeutic target have been recognized in many diseases such as cancer, autoimmune and neurodegenerative disorders (Bonam et al., 2019). In contrast, the exploration of lysosome-targeted therapeutics in skeletal diseases has been limited. In addition to exploring druggable target among lysosome proteins, a profound comprehension of lysosomal proteins and autophagosome-lysosome fusion is instrumental in elucidating the pathogenesis and disease progression, thereby facilitating the development of therapeutic interventions.

### 7.1 Osteopetrosis:advancing gene therapy for improved treatment

Osteopetrosis is a hereditary disease characterized by high bone mass and increased bone fragility. It is known that the mutations in *TCIRG1, CLCN7, OSTM1, SNX10* or *PLEKHM1* can result in autosomal recessive osteopetrosis with abundant but non-functional OCs. Interestingly, despite the proposed functions of these genes, OCs from affected individuals exhibited normal actin-ring formation and acidic microenvironments *in vitro*, but displayed inhibited RB formation, suggesting a potential impact on vesicular trafficking mechanisms regulated by these genes (Sobacchi et al., 2013). Autophagy deficiency has been observed in auto recessive osteopetrosis mice carrying mutations in TCIRG1, potentially resulting in compromised degradation of autophagic cargo (Wang et al., 2023). The diverse clinical manifestations associated with different gene mutations provide valuable information for tailoring personalized treatment strategies. Individuals with *CLCN7* mutations commonly suffer from latent neurodegeneration within the initial 3–6 months of life, a condition unresponsive to hematopoietic stem cell transplantation (HSCT), the current definitive therapeutic option, necessitating ongoing vigilant monitoring (Sobacchi et al., 2013). In comparison to HSCT, gene therapy utilizing autologous hematopoietic stem and progenitor cells presents potential advantages over HSCT due to its ability to be promptly arranged following diagnosis, thereby optimizing therapeutic outcomes and decreasing the occurrence of graft-versus-host diseases and other potential transplant-related complications. However, despite these potential benefits, gene therapy using autologous hematopoietic stem and progenitor cells has not yet been implemented in clinical practice (Moscatelli et al., 2021; Chen et al., 2019). Consequently, further research efforts should be directed towards elucidating the molecular mechanisms underlying gene mutations and their impact on clinical phenotypic alterations, including the intricate regulatory mechanisms and interactions among various proteins.

### 7.2 Osteoporosis: lysosomes-related therapeutic targets for OC regulation

Osteoporosis is a chronic skeletal disorder characterized by reduced bone density, compromised microstructure of bone tissue, heightened bone fragility, and elevated susceptibility to fractures. This condition arises from the dysregulation of bone homeostasis, wherein the process of bone resorption by OCs surpasses bone formation by OBs (Li et al., 2023; Foessl et al., 2023). Targeting lysosomal proteins, which play a crucial role in various stages of OC activity, may offer a potential therapeutic approach to mitigate the excessive resorptive function of OCs or inhibit osteoclastogenesis. Some of these treatments had been put into clinical trial. For example, Odanacatib, an oral inhibitor of CTSK, has demonstrated the ability to improve bone mineral density over a 5-year period, strengthen bones, and reduce the incidence of fractures in both the vertebral and nonvertebral regions. However, its was discontinued in the Long-term Odanacatib Fracture Trial due to adverse effects, including strokes (Chapurlat, 2015; Khosla and Hofbauer, 2017). Moreover, research in animal models and cell studies suggested that targeting V-ATPase may offer a promising approach for developing new anti-resorptive therapies for osteoporosis. Luteolin, an inhibitor specific to OCs that targets the interaction between V-ATPase a3-d2, effectively inhibits osteoclastic resorptive activity without compromising osteoclastogenesis (Chu et al., 2021; Shin et al., 2012). This preservation of OC-OB coupling promotes osteoblastic bone formation (Teitelbaum, 2016). However, it remains uncertain whether these inhibitors can maintain their viability with appropriate selectivity and minimized potential adverse effect in clinical application. Furthermore, the precise functions of a majority of V-ATPase isoforms in OCs are not fully elucidated yet, necessitating further in-depth research. Targeting autophagy via inhibiting fusion of autophagosome and lysosome in OCs may also be feasible in treating the fast bone loss caused by excessive osteoclastic bone resorption. Studies have shown that the autophagy inhibitor chloroquine can effectively impede osteoclastogenesis, mitigate OC activation induced by ovariectomy, and consequently prevent bone loss *in vivo*. Chloroquine has been reported to mainly block autophagic flux by inhibiting the autogosome fusion with lysosomes, and more precisely, the transport of cargo targeted for degradation to lysosomes in other cell types. More related studies are yet to be established (Mauthe et al., 2018; Li et al., 2020).

## 8 Summary and future perspectives

The biogenesis of lysosomes is a complicated process which is closely associated with various organelles, such as endoplasmic reticulum, Golgi apparatus and plasma, and is regulated by multiple transcription factors. We select crucial process to discuss the biogenesis of lysosomes, including the biosynthesis and delivery of lysosomal hydrolases, endosome-lysosome trafficking, lysosomal fusion and reformation, and cargo degradation. Other process, such as lysosome repair and clearance, which is involved in maintaining lysosomal pool homeostasis, or lysosome exocytosis, which may play an important role in immune responses, also require further research. Moreover, apart from the M6P pathway, the mechanisms regulating the sorting of lysosomal proteins have not been fully elucidated.

Lysosomes, as metabolic signaling hubs, are intricately linked to OC differentiation. Despite the identified correlation of many lysosomal related factors with OC differentiation, few studied have down to explore the possibility to inhibit OC differentiation by blocking lysosomal biogenesis. In addition, we also present the regulation of lysosome involved in the bone resorption and OC autophagy. On the one hand, lysosomes serve as the regulator of formation and maintenance of the resorptive organelle, RB, as well as the supplier of protein required for degradation of organic and inorganic bone matrix during bone resorption. The whole process of lysosome biogenesis and vesicular trafficking is regulated by several factors, although the specific function of many of them remain implicit. Meanwhile, as an essential part of vesicle trafficking, transcytosis pathway by secretory lysosome has not been fully understood, which offers new perspectives for future research. Furthermore, the interaction of Rab7 and V-ATPase and indirect activation of proteolytic enzymes by lacunae acidification protein suggest the potential network between the protein groups in the process of bone resorption. Meanwhile, the protein groups are not limited in bone resorption, which means they play multiple roles in OCs including OC differentiation and autophagy. A closer look of interaction mechanism among proteins in different stages of OCs contributes to better treatment of bone resorption related diseases. On the other hand, lysosomes are considered as the core of degradation of damaged or senescent organelles during autophagy. The OC autophagy is divided into five steps (Chen et al., 2023): initiation, nucleation, elongation, maturation, and degradation, among which the fusion between autophagosome and lysosome is the major event. However, current research on the autophagosome-lysosome fusion in OCs is limited, so it is advisable to further explore this process in future. Additionally, autophagy may be closely associated with OC differentiation, migration and function. Interestingly, over-activated autophagy may accelerate OC apoptosis and senescence (Wang T. et al., 2020), suggesting how to regulate precisely the degree of OC autophagy might be the new direction for the treatment of OC-related disorders.

In summary, current research findings support the essential role of lysosomes in OCs. Further exploration of lysosome-related pathways in OCs has the potential to promote the development of lysosomal targeted therapies that selectively inhibit OCs and effectively treat bone disorders associated with OC dysfunction.

## Figures and Tables

**Table:** 

**BL**	basolateral membrane
**Ca** ^**2+**^	calcium ions
**[Ca**_**3**_ **(PO4)**_**2**_ **]**_**3**_ **Ca(OH)**_**2**_	crystalline hydroxyapatite
**CLEAR**	Coordinated lysosomal expression and regulation
**ClC-7**	chloride channel type 7
**CTSK**	cathepsin K
**FSD**	functional secretory domain
**GEF**	guanine nucleotide exchange factor
**GSK-3β**	glycogen synthase kinase 3 beta
**H4K16**	histone H4 lysine 16 residue
**HSCT**	hematopoietic stem cell transplantation
**LAMP**	lysosome-associated membrane proteins
**LMPs**	lysosomal membrane proteins
**MMP**	matrix metalloproteinase
**MPR**	mannose 6-P receptor
**mTORC1**	mechanistic target of rapamycin complex 1
**NFATc1**	nuclear factor of activated T cells 1
**OCs**	Osteoclasts
**OBs**	Osteoblasts
**Ostm1**	osteoclastogenesis associated transmembrane protein 1
**PtdIns(3,5) P2**	phosphatidylinositol 3,5-bisphosphate
**PtdIns3P**	phosphatidylinositol 3-phosphate
**PLEKHM1**	protein family member 1
**RILP**	Rab interacting lysosomal protein
**RB**	ruffled border
**SZ**	sealing zone
**STAT3**	signal transducer and activator of transcription 3
**SNX**	sorting nexin
**TET2**	Tet methylcytosine dioxygenase 2
**TFEB**	transcription factor EB
**TFE3**	transcription factor E3
**TRAP**	tartrate-resistant acid phosphatase
**TRPML1**	transient receptor potential mucolipin 1
**TGN**	trans-Golgi network
**V-ATPase**	vacuolar-type ATPase.
